# Hybrid approach to closure in fetoscopic myelomeningocele repair

**DOI:** 10.3171/2026.1.FOCVID25222

**Published:** 2026-04-01

**Authors:** Natasha Hongsermeier-Graves, Vance R. Mortimer, Stephen J. Fenton, Martha A. Monson, Robert J. Bollo

**Affiliations:** 1 Department of Neurosurgery, Clinical Neurosciences Center, University of Utah, Salt Lake City;; 2 Department of Surgery, Division of Pediatric Surgery, University of Utah School of Medicine and Primary Children’s Hospital, Salt Lake City;; 3 Department of Obstetrics and Gynecology, Division of Maternal-Fetal Medicine, University of Utah, Salt Lake City; and; 4 Department of Neurosurgery, Division of Pediatric Neurosurgery, University of Utah School of Medicine and Primary Children’s Hospital, Salt Lake City, Utah

**Keywords:** fetoscopic, fetal surgery, myelomeningocele, myeloschisis, neural tube defect, spina bifida

## Abstract

Neural tube defects (NTDs) affect 2 in 1000 births worldwide. Myelomeningocele (MMC) represents a severe NTD, often with permanent neurological sequelae. The randomized multicenter Management of Myelomeningocele Study (MOMS) showed that open prenatal (compared to postnatal) myelomeningocele closure could reverse hindbrain herniation, lower rates of shunt insertion for hydrocephalus, and improve neurological function, with the trade-off of increased maternal complications and premature birth. Fetoscopic approaches may have equivalent benefits to open fetal MMC closure with fewer maternal complications. The hybrid approach to fetoscopic repair (primary closure vs biosubstitute patch) described in this video can be tailored to individual fetal anatomy and promote comparable epithelialization over time.

The video can be found here: https://stream.cadmore.media/r10.3171/2026.1.FOCVID25222

## Transcript

### 0:24 Background.^1–5^

Neural tube defects affect approximately 2 in 1000 births worldwide, with myelomeningocele representing its most severe form. Prenatal repair has been shown to portend better neurological outcomes even despite increased maternal complications and premature birth. Minimally invasive fetoscopic approaches likely extend the benefits of prenatal repair while reducing risks. Here we show our hybrid approach to fetoscopic closure with primary closure versus an acellular dermal matrix patch.

### 0:55 Patient Positioning.

Not shown, the adult patient was positioned supine in dorsal lithotomy position. Maneuvers, when needed, are used to place the fetal patient prone and cephalic, with the head positioned in the pelvis.

### 1:07 Laparotomy and Fetoscopic Hysterotomy.

A midline laparotomy is created from just above the umbilicus to the pubis in order to gain access to the uterus. The uterus is then externalized and subsequently accessed using three fetoscopic ports.

### 1:18 Dissection at the Transitional Zone.

After identifying the area of myeloschisis, the placode is dissected from abnormal skin via the surrounding transitional arachnoid plane circumferentially, with care to identify and preserve spinal cord tissue and ventral nerve roots. At the conclusion of this step, the placode can now retract back into the spinal canal.

### 1:41 Dissection at the Junctional Zone.

The skin is then dissected widely, again circumferentially, in the suprafascial plane between the skin and the lumbodorsal fascia. Skin may be opened in the midline to obtain better exposure.

### 1:59 Dissection of Myofascial Flaps.

Myofascial flaps are raised for coverage of the placode. This is done using electrocautery starting out laterally on both sides, then moving cranially and caudally until the myofascial flaps are mobile and easily brought to the midline, as well as long enough to cover the defect.

### 2:22 Closure of Myofascial Flaps.

Barbed 3-0 running V-Loc sutures are used to close the myofascial flaps over the placode and its overlying tissue graft for a watertight closure. Of note, the placode is first covered by an otologic porcine graft before bringing the myofascial flaps together over top. A suture is used at both the cranial and caudal ends, then tied together in the middle.

### 2:51 Closure of Skin.^6^

Skin flaps are closed in the midline using a barbed 4-0 V-Loc suture with an acellular dermal matrix, such as AlloDerm, underlay when primary closure is achievable with minimal tension. When this is not possible, the AlloDerm can be incorporated as a patch to minimize tension on the skin closure. In this case, the edge of the AlloDerm is sewn to the skin edges. When AlloDerm is used only as an underlay, however, the skin edges are brought together primarily over the underlay without securing the AlloDerm to the skin. By adopting a hybrid approach that is flexible based on individual fetal anatomy, the use of AlloDerm (either under a primary closure or within the skin closure layer) promotes optimal wound healing and epithelialization over time. A suture is used at both the cranial and caudal ends, then tied together in the middle. First, we show a primary closure with AlloDerm underlay. Second, we show incorporation of a patch. In one place, the AlloDerm patch is visible from the outside, whereas the remainder of the skin has been closed primarily.

### 4:00 Closure of Hysterotomy.

After evacuating insufflation gas, infusing warm LR (lactated Ringer’s solution) back to the preoperative amniotic fluid volume, and administering intrauterine nafcillin, trocars are removed and previously placed sutures are tied to close the defects. The uterus is returned to the abdomen, and the laparotomy is closed in layered fashion.

## Disclosures

The authors report no conflict of interest concerning the materials or methods used in this study or the findings specified in this publication.

## Author Contributions

Primary surgeon: Bollo, Fenton. Assistant surgeon: Monson. Editing and drafting the video and abstract: Bollo, Hongsermeier-Graves, Mortimer, Monson. Critically revising the work: Bollo, Hongsermeier-Graves, Monson. Reviewed submitted version of the work: Hongsermeier-Graves, Fenton, Monson. Approved the final version of the work on behalf of all authors: Bollo. Supervision: Bollo, Monson.

## Supplemental Information

### Patient Informed Consent

The necessary patient informed consent was obtained in this study.
